# Endogenous Bok is stable at the endoplasmic reticulum membrane and does not mediate proteasome inhibitor-induced apoptosis

**DOI:** 10.3389/fcell.2022.1094302

**Published:** 2022-12-19

**Authors:** Caden G. Bonzerato, Katherine R. Keller, Jacqualyn J. Schulman, Xiaokong Gao, Laura M. Szczesniak, Richard J. H. Wojcikiewicz

**Affiliations:** Department of Pharmacology, SUNY Upstate Medical University, Syracuse, NY, United States

**Keywords:** Bcl-2 related ovarian killer, B cell Lymphoma 2 (Bcl-2) family, ER membrane, myeloid-cell leukemia 1, apoptosis, ubiquitin proteasomal pathway, inositol 1,4,5-trisphosphate receptor

## Abstract

Controversy surrounds the cellular role of the Bcl-2 family protein Bok. On one hand, it has been shown that all endogenous Bok is bound to inositol 1,4,5-trisphosphate receptors (IP_3_Rs), while other data suggest that Bok can act as a pro-apoptotic mitochondrial outer membrane permeabilization mediator, apparently kept at very low and non-apoptotic levels by efficient proteasome-mediated degradation. Here we show that 1) endogenous Bok is expressed at readily-detectable levels in key cultured cells (e.g., mouse embryonic fibroblasts and HCT116 cells) and is not constitutively degraded by the proteasome, 2) proteasome inhibitor-induced apoptosis is not mediated by Bok, 3) endogenous Bok expression level is critically dependent on the presence of IP_3_Rs, 4) endogenous Bok is rapidly degraded by the ubiquitin-proteasome pathway in the absence of IP_3_Rs at the endoplasmic reticulum membrane, and 5) charged residues in the transmembrane region of Bok affect its stability, ability to interact with Mcl-1, and pro-apoptotic activity when over-expressed. Overall, these data indicate that endogenous Bok levels are not governed by proteasomal activity (except when IP_3_Rs are deleted) and that while endogenous Bok plays little or no role in apoptotic signaling, exogenous Bok can mediate apoptosis in a manner dependent on its transmembrane domain.

## Introduction

Bcl-2-related ovarian killer (Bok) is a member of the Bcl-2 protein family network that helps control cell viability (Moldoveanu et al., 2014; Kale et al., 2018; Kalkavan and Green, 2018), but its place in that network and its role within the cell remains unclear and controversial (D'Orsi et al., 2017; Joshi et al., 2020; Naim and Kaufmann, 2020; Shalaby et al., 2020; Means and Katz, 2021). On one hand, it has been shown that Bok over-expression in mammalian cells can trigger apoptosis (Echeverry et al., 2013; Einsele-Scholz et al., 2016; Llambi et al., 2016; Stehle et al., 2018) and that purified recombinant Bok can permeabilize liposomes (Llambi et al., 2016; Fernandez-Marrero et al., 2017; Zheng et al., 2018; Shalaby et al., 2022), indicating that Bok may trigger mitochondrial outer membrane permeabilization (MOMP), similarly to the better-characterized pro-apoptotic proteins Bak and Bax (Moldoveanu et al., 2014; Kale et al., 2018; Kalkavan and Green, 2018; Pena-Blanco and Garcia-Saez, 2018). Further, it has been proposed that Bok is constitutively pro-apoptotic, with Bok expression maintained at very low and “safe” levels by activity of the ubiquitin-proteasome pathway (UPP) (Llambi et al., 2016), and that Bok accumulates and causes cell death only if its UPP-dependent processing is blocked with proteasome inhibitors (Llambi et al., 2016; Naim and Kaufmann, 2020; Shalaby et al., 2020). On the other hand, it appears that endogenous Bok is constitutively bound to endoplasmic reticulum (ER)-localized inositol 1,4,5-trisphosphate receptors (IP_3_Rs) and thus may not be free to translocate to mitochondria to cause MOMP (Schulman et al., 2013; Schulman et al., 2016; Schulman et al., 2019; Szczesniak et al., 2021a). Further, it has been reported that Bok is enriched in mitochondria-associated membranes (MAMs), where it appears to maintain MAM integrity and facilitate efficient transfer of Ca^2+^ from the ER to mitochondria (Carpio et al., 2021) and perhaps participate in ER-stress signaling (Walter et al., 2022).

Here we report that under standard conditions, endogenous Bok is present at readily-detectable levels in key cultured cell lines, that its levels are not governed by the UPP, and that it does not mediate proteasome inhibitor-induced apoptosis. However, endogenous Bok levels do fall dramatically when IP_3_Rs are deleted, and under such conditions, Bok is still localized to the ER membrane and is rapidly degraded by the UPP. Finally, the unique, positively-charged residues in the transmembrane (TM) domain of Bok regulate its stability, ability to interact with Mcl-1 and, when over-expressed, its ability to trigger apoptosis.

## Materials and methods

### Materials

Mouse embryonic fibroblasts (MEFs) (Karbowski et al., 2006), mouse pituitary αT3 cells and monkey kidney Cos-7 cells were maintained at 37°C under 5% CO_2_ in Dulbecco’s modified Eagle’s medium supplemented with 5% fetal bovine serum, 100 U/ml penicillin, and 100 μg/ml streptomycin. Human embryonic kidney (HEK293T), human colon carcinoma (HCT116) (Wang and Youle, 2012), and human HeLa cells were maintained identically, except with 10% serum. Rabbit antibodies used were: anti-IP_3_R1, anti-IP_3_R2 (Wojcikiewicz, 1995), anti-erlin2, anti-gp78, anti-Hrd1 (Pearce et al., 2007), anti-Mcl-1 #D35A5, anti-Bcl-2 #D17C4, anti-caspase-3 #9662, anti-Bak #12105, anti-Bax #2772, anti-LC3A/B #4108, anti-PARP #9542, anti-caspase-8 #8592 (Cell Signaling Technology), anti-Bok^A^ (raised against amino acids 19–32 of mouse Bok) (Ke et al., 2012; Echeverry et al., 2013), anti-Bok^B^ (Abcam # 186745, raised against amino acids 1–150 of human Bok), and anti-transaldolase (Pearce et al., 2007). Mouse monoclonal antibodies used were: anti-CHOP #7351, anti-ube2J1 #100624 (Santa Cruz Biotechnology Inc.), anti-ubiquitin clone FK2 (BioMol), anti-FLAG epitope clone M2 #F3165 (Sigma), anti-HA epitope clone HA11 (Covance), anti-IP_3_R3 #610313 (BD Transduction Labs), anti-p97 #10R-P104A (Fitzgerald), anti-cytochrome c #13575 and anti-complex V/III #14748/14745 (Abcam). Horseradish peroxidase-conjugated secondary antibodies, protease inhibitors, Triton X-100, CHAPS, brefeldin A (BFA), tunicamycin (TN), thapsigargin (TG), bafilomycinA1 (BAFA1), and cycloheximide (CHX) were purchased from Sigma. MG132 and staurosporine (SST) were from Enzo Life Sciences. Q-VD-OPH and CB-5083 were from Cayman Chemical. Protein A-Sepharose CL-4B was from GE Healthcare. Linear, MW∼25,000 polyethylenimine (PEI) was from Polysciences Inc. Precision Plus™ Protein Standards, and SDS-PAGE reagents were from Bio-Rad. TAK-243 was from MedChemExpress. Lipofectamine 2000 and Opti-MEM were from ThermoFisher. Trypsin-EDTA and Trypan Blue were from Corning.

### Cell lysis, immunoprecipitation (IP), and immunoblotting

Cell lysates and IPs were prepared as described (Schulman et al., 2019). Mouse brain, rat brain, and human kidney (obtained from the Upstate Biorepository Center) were prepared as described (Schulman et al., 2016). All samples were resuspended in gel-loading buffer (Oberdorf et al., 1999), incubated at 37°C for 30 min, subjected to SDS-PAGE, and proteins were transferred to nitrocellulose for probing as described (Schulman et al., 2019). Immunoreactivity was detected using Pico Chemiluminescent Substrates (ThermoFisher #34579) and a ChemiDoc imager (Bio-Rad).

### Generation of CRISPR/Cas9-mediated knockout (KO) cell lines

The CRISPR/Cas9 system was used to target exons within the Bok, IP_3_R1, IP_3_R2, ube2J1, gp78, erlin2, and Hrd1 genes using the gRNAs indicated (Supplementary Table S1), with constructs introduced by Neon transfection (Invitrogen; 10 µg total DNA per 3 × 10^6^ cells in 100 μl, 1 pulse, 20 ms, 1,500 V). As previously described (Schulman et al., 2013), IP_3_R1 was targeted in MEFs using either gRNA 1 or 2 together with vectors encoding hCas9 (Addgene) and enhanced green fluorescent protein (EGFP) (Clontech). IP_3_R2 was then targeted in wild-type and IP_3_R1 KO MEFs using gRNA 1 or 2 in the pCas-Guide-EF1a-GFP vector (Origene) to generate IP_3_R2 KO and IP_3_R1/2 KO MEFs. Bok was targeted using gRNA 1 or 2 (Schulman et al., 2019) in wild-type and Bak/Bax double KO MEFs (DKO MEFs) (Karbowski et al., 2006) together with vectors encoding hCas9 and the Clover EGFP mutant (Addgene) to generate Bok KO MEFs (BKO MEFs) and Bak/Bax/Bok triple KO MEFs (TKO MEFs). Ube2J1, gp78, erlin2, and Hrd1 were targeted in IP_3_R1/2 KO MEFs using gRNAs in the pCas-Guide-EF1a-GFP vector (Origene). In all cases, fluorescent protein-expressing cells were isolated 48 h post-transfection by fluorescence-activated cell sorting (FACS) and plated at 1 cell/well in 96-well plates. Colonies were expanded and screened in immunoblots for loss of target protein. Multiple independent cell lines from each gRNA were used for all experiments. In some experiments (Figure 2B), sorted cells were plated at 10,000 cells/9.6 cm^2^ well, which were expanded and analyzed as “heterogenous cultures.”

### Analysis of exogenous Bok constructs *via* transient transfection

Mouse Bok tagged at the N-terminus with a triple FLAG epitope (3F-Bok) (Schulman et al., 2016) was used to generate 3F-Bok^6KR^, 3F-Bok^K160R^, and 3F-Bok^K160A^ by mutagenic PCR. HEK293T and Bak/Bax KO double (DKO) HCT116 cells (Wang and Youle, 2012) seeded at 6 × 10^5^/9.6 cm^2^ well were transfected ∼24 h later with 0.125 µg of Bok cDNAs and 6 µl of 1 mg/ml PEI (pre-mixed in 50 µl of serum-free cultured medium), and ∼24 h later were harvested with ∼0.2 ml/well lysis buffer and analyzed *via* immunoblotting. Mouse 3F-Bok^R200A/K203A^ and 3F-Bok^Δ^™ (amino acids 1–187 of mouse Bok) were generated as described (Schulman et al., 2016; Szczesniak et al., 2021a). MEFs transfected with 3F-Bok constructs and mouse IP_3_R1HA (Szczesniak et al., 2021a; Gao et al., 2022) using the Neon Transfection system described above were seeded at 6 × 10^5^/9.6 cm^2^ well and 48 h post-transfection, cells were harvested with ∼0.1 ml/well lysis buffer. HeLa Bok KO cells seeded at 3 × 10^5^/9.6 cm^2^ well were transfected with mouse 1F-Mcl-1, 3F-Bok^WT^, 3F-Bok^R200A/K203A^ or 3F-Bok^Δ^™ using 5 µl of Lipofectamine 2000 and ∼24 h later were harvested with ∼0.1 ml/well lysis buffer. The authenticity of all cDNAs was confirmed by DNA sequencing (Genewiz).

### Measurement of cell death

Cells were seeded at 3 × 10^5^/9.6 cm^2^ well and were transfected with 3F-Bok^WT^ or 3F-Bok^R200A/K203A^, or were treated with 10 µM of MG132 for various times. Cells were then harvested with 0.5 ml trypsin-EDTA and then mixed with trypan blue at a final concentration of 0.2% w/v for 10 min. Cells were counted with a TC20 Automated Cell Counter (Bio-Rad) and cell death was measured by the inability to exclude trypan blue.

### Generation of αT3 cells stably expressing IP_3_Rs

As previously described (Wright et al., 2018), αT3 IP_3_R1 KO cells (Schulman et al., 2016) were transfected to express mouse IP_3_R1HA^WT^ and IP_3_R1HA^Δ^ ^1916/17^ using the Neon Transfection System followed by selection in 1.3 mg/ml G418 for 72 h. Cells were then plated at 1 cell/well in 96-well plates, expanded, screened in immunoblots for IP_3_R1 expression, and maintained in 0.3 mg/ml G418. Multiple clones were characterized in all experiments.

### Measurement of Bok mRNA levels

RNA was extracted from 10^6^ MEFs using the RNeasy Mini Kit (Qiagen) and reverse transcribed into cDNA using the iScript cDNA Synthesis Kit (Bio-Rad). qRT-PCR for Bok mRNA levels was performed using the forward primer GAT​GGA​CGG​ATG​TCC​TCA​AG and the reverse primer TCT​CTG​GCA​ACA​ACA​GGA​AG (Schulman et al., 2016) with SsoAdvanced Universal SYBR^®^ Green Supermix, a CFX Connect Real-Time PCR Detection System thermal cycler (Bio-Rad), and the following PCR cycling parameters: 30 s at 95°C and then 40 cycles of 95°C for 15 s and 60°C for 30 s. Results were analyzed by normalizing to the housekeeping genes S18, peptidylpropyl isomerase A, and hypoxanthine phosphoribosyl-transferase 1, using the Bio-Rad CFX Maestro Software and the 2^−ΔΔCT^ method (Schulman et al., 2016).

### Subcellular fractionation

Cell fractionation was performed using WT and IP_3_R1/2 KO MEFs essentially as described (Wieckowski et al., 2009; Morciano et al., 2016) with all steps at 4°C. Approximately 10^8^ cells were collected in 155 mM NaCl, 10 mM HEPES, 1 mM EDTA, pH 7.4, and centrifuged at 500 × g for 5 min. Cell pellets were then washed with phosphate-buffered saline and re-centrifuged at 500 × g for 5 min. Cell pellets were then resuspended with 10 ml homogenization buffer (225 mM mannitol, 75 mM sucrose, 30 mM Tris-HCl, 0.1 mM EGTA, 10 μM pepstatin, 0.2 mM phenylmethylsulfonyl fluoride, 1 mM dithiothreitol, and 0.2 μM soybean trypsin inhibitor, pH 7.4) and disrupted using a Kinematica Polytron^®^ PT 3100 Homogenizer for 5–10 s to obtain ∼70% plasma membrane disruption as indicated by trypan blue. The homogenized cells were centrifuged at 600 × g for 5 min to remove nuclei and unbroken cells. The supernatant (Homogenate, H) was then centrifuged at 1,800 × g for 10 min to pellet the crude mitochondria (CM). The subsequent supernatant was then centrifuged at 100,000 × g for 90 min using a Beckman Optima™ L-90K Ultracentrifuge (70-Ti rotor) to isolate the ER (pellet) and the cytosol, C (supernatant). The CM and ER pellets were resuspended in 10 ml homogenization buffer and subjected to SDS-PAGE along with H and C fractions.

### Data analysis

All experiments were repeated two or more times (*n* = the number of independent experiments) and representative images of immunoblots and traces are shown. Quantitated data are expressed as mean ± SEM. Immunoreactivity quantification was performed using ImageLab software (BioRad). The appropriate bands were highlighted and volume intensity (high signal/noise ratio) or adjusted volume intensity (low signal/noise ratio) was measured in arbitrary units, A.U. Statistical analysis was performed using a Student’s *t*-test (with Welch’s correction in Figure 7) and *p*-values of <0.05 and <0.005 were considered statistically significant and denoted with “*” and “**” respectively, while *p*-values of >0.05 were not considered statistically significant and denoted with “#”.

## Results

### Endogenous Bok is expressed at readily-detectable levels in various cell types

**FIGURE 1:**
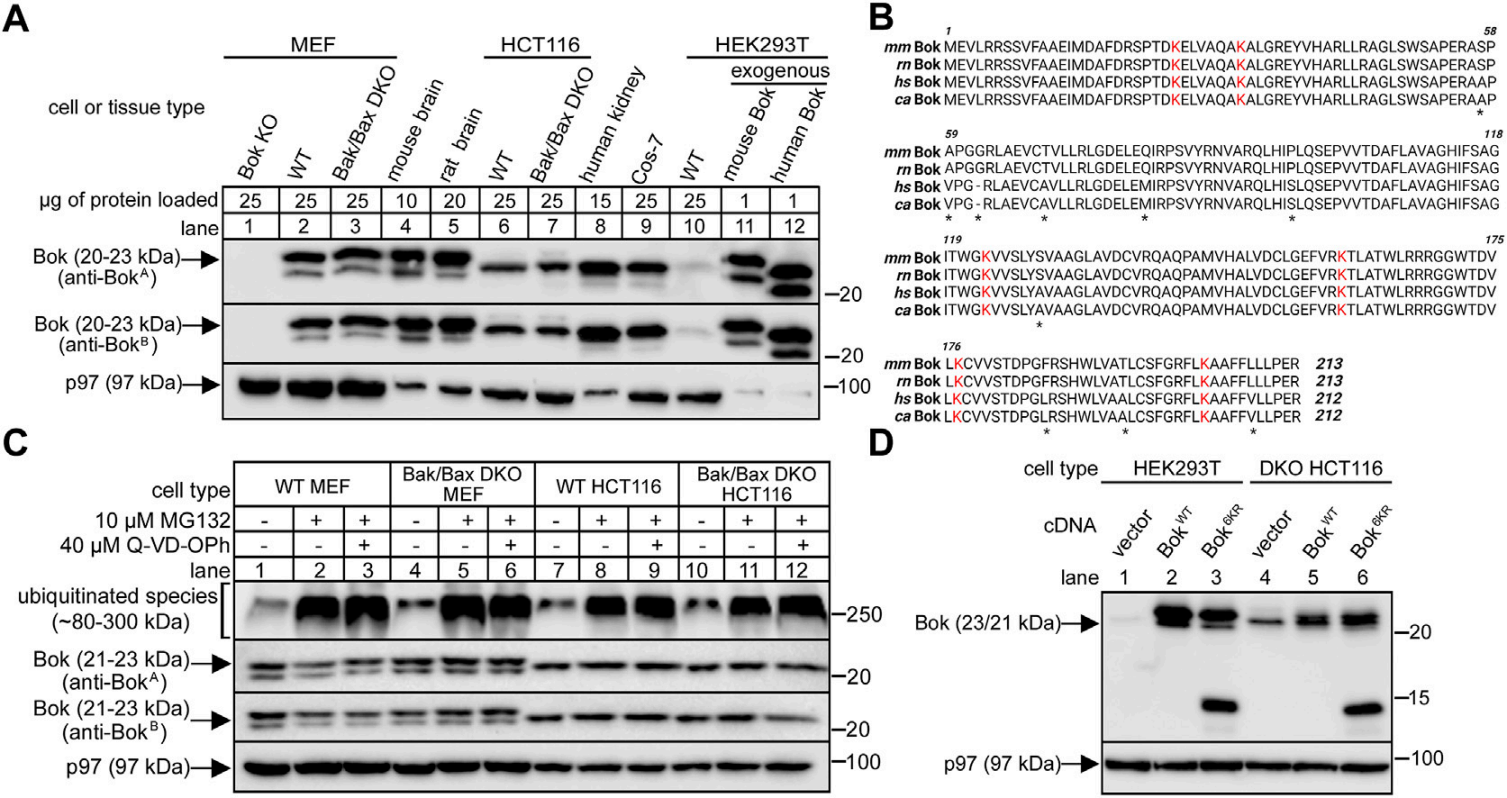
Bok expression in various cell lines and tissues and insensitivity to proteasome inhibition or lysine mutation. **(A)**, Endogenous Bok immunoreactivity in lysates from various cell lines and tissues, with p97 serving as a loading control. Anti-Bok^A^ is raised against amino acids 19–32 of mouse Bok and anti-Bok^B^ is raised against amino acids 1–150 of human Bok. Different amounts of protein were loaded to optimize visualization of immunoreactivity. Note that Bok migrates as a 23/21 kDa doublet in mouse and rat samples, and predominantly at 22 kDa in human samples. **(B)**, Amino acid sequences of mouse (mm), rat (rn), human (hs) and monkey (cs) Bok (accession numbers O35425, Q792S6, Q9UMX3, and A0A0D9R0C9, respectively), with asterisks denoting sequence differences and with lysine residues highlighted in red. **(C)**, Endogenous Bok immunoreactivity in lysates from MEF and HCT116 cells incubated without or with 10 μM MG132 and 40 μM Q-VD-OPh for 8 h, with p97 serving as a loading control and ubiquitinated species serving as a positive control for MG132 effectiveness. **(D),** Effect of lysine mutation on exogenous Bok expression. HEK or DKO HCT116 cells were transiently transfected to express mouse Bok^WT^ or Bok^6KR^, in which all six lysines are mutated to arginine, and cell lysates were probed as indicated with anti-Bok^A^, with p97 serving as a loading control. While Bok^WT^ migrates at 23/21 kDa, Bok^6KR^ was partially fragmented, as indicated by the appearance of additional bands at 14 and 12 kDa.

It has been reported that Bok expression in cell lines is highly variable (e.g., undetectable in MEFs, but relatively high in HCT116 cells), and that Bok expression is strongly enhanced by proteasome inhibition (Llambi et al., 2016). Since this has not been universally observed and indeed, proteasome inhibition often does not affect Bok levels (Echeverry et al., 2013; Schulman et al., 2013; Carpio et al., 2015; D'Orsi et al., 2016; Schulman et al., 2016; Moravcikova et al., 2017; Schulman et al., 2019), we sought to clarify the situation by re-examining Bok levels in various key cell lines with multiple Bok antibodies. We find that Bok is readily-detectable at 23/21 kDa in MEFs; both in WT MEFs and in MEFs lacking Bak/Bax (double knockout “DKO” MEFs), with both anti-Bok^A^ and anti-Bok^B^ (Figure 1A, lanes 2 and 3). Lack of immunoreactivity in Bok KO MEFs (Schulman et al., 2019) served as a negative control for the antibodies, showing that they specifically recognize Bok (Figure 1A, lane 1). We have shown previously that other mouse cell lines and tissues express 23/21 kDa Bok, and that the two species result from the use of alternative translation start sites at codons for Met1 and Met15 (Schulman et al., 2016). Likewise, identical to mouse tissue (Figure 1A, lane 4), rat tissue expresses 23/21 kDa Bok (lane 5). Readily-detectable levels of Bok immunoreactivity were also seen in WT HCT116 cells and in HCT116 cells lacking Bak/Bax (DKO HCT116 cells), although therein Bok migrated predominantly at 22 kDa (lanes 6 and 7). Bok also migrates predominantly at 22 kDa in human tissue and monkey Cos-7 cells (with also a weak immunoreactive band at 20 kDa), and in HEK293T cells, in which Bok immunoreactivity was particularly low (lanes 8–10). The discrepancy between the apparent size of mouse and human Bok was also seen when they were expressed from cDNAs in HEK293T cells; exogenous mouse and human Bok migrated at 23/21 kDa and 22/20 kDa, respectively (lanes 11 and 12), indicating that both have the capacity to utilize alternative translation start sites. The reason for the migration discrepancy is surprising given that the mouse/rat versus human/monkey amino acid sequences are 95% identical (Figure 1B), but is presumably accounted for by slight sequence variations (e.g., glycine vs. no glycine at position 62 and proline vs. serine at position 96).

Also, in contrast to previous findings (Llambi et al., 2016), we observed that proteasomal inhibition did not alter endogenous Bok levels, since Bok immunoreactivity measured with both anti-Bok^A^ and anti-Bok^B^ was largely unaltered by treatment of cells with the proteasome inhibitor MG132, in the absence or presence of the pan-caspase inhibitor Q-VD-OPh, which should protect cells against potential apoptosis if Bok was elevated (Figure 1C). Further, we did not observe an increase in the expression level of exogenous Bok when all of the six lysine residues that could potentially serve as ubiquitination sites (Figure 1B) were blocked by mutation to arginine (Figure 1D). In fact, and very surprisingly, the lysine-free Bok mutant was partially fragmented (Figure 1D, lanes 3 and 6), for a reason that can be attributed to mutation of K160 to arginine (Supplementary Figure S1). While the reason for the discrepancy between our findings and those previously reported (Llambi et al., 2016) are unclear, the notion that cell survival is dependent upon constitutive ubiquitination and proteasomal degradation of Bok to vanishingly low levels appears to require re-evaluation.

### Endogenous Bok does not mediate proteasome inhibitor-induced apoptosis in MEFs

**FIGURE 2:**
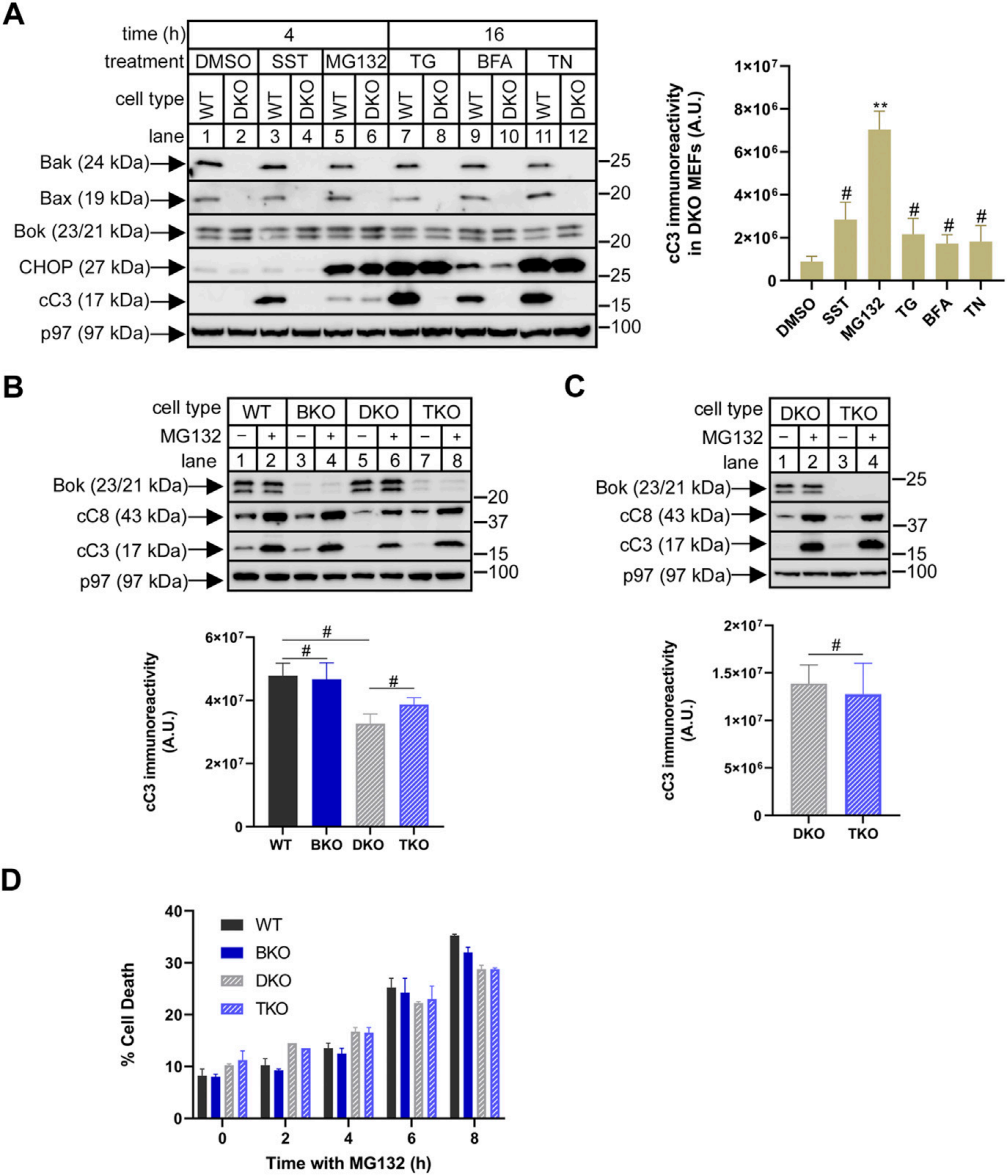
Bok does not mediate MG132-induced apoptosis in Bak/Bax-deficient MEFs. **(A)**, WT and Bak/Bax double-knockout (DKO) MEFs (odd and even numbered lanes, respectively) were incubated as indicated with DMSO (vehicle), 1 µM staurosporine (SST), 10 µM MG132, 1 µM thapsigargin (TG), 0.5 μg/ml brefeldin A (BFA), or 0.5 μg/ml tunicamycin (TN), and cell lysates were probed in immunoblots for the proteins indicated, with p97 serving as a loading control and CHOP as a marker of ER stress. The histogram shows quantitated cleaved caspase-3 (cC3) immunoreactivity in DKO MEFs (mean ± SEM *n* = 4, ** designates *p* < 0.005 and # designates not significant, *p* > 0.05, for responses to SST, MG132, TG, BFA, and TN vs. DMSO control). **(B,C)**, Using CRISPR/Cas9, Bok was deleted from either WT or DKO MEFs, creating Bok KO (BKO) and triple KO (TKO) MEFs. Heterogeneous cultures **(B)**, or cloned cell lines **(C)**, were incubated as indicated without or with 10 µM MG132 for 4 h and cell lysates were probed in immunoblots for the proteins indicated, with p97 serving as a loading control. The histograms show quantitated cC3 immunoreactivity for MG132-treated cells (mean ± SEM, *n* = 2 **(B)**, and *n* = 3 **(C)**; # designates not significant, *p* > 0.05). **(D)**, Cell death in heterogenous cultures incubated with 10 µM MG132, assessed by the inability to exclude trypan blue (mean ± SEM, *n* = 2).

Because Bok is well-expressed under normal conditions (Figure 1A), we examined whether it might indeed play a role in mediating the pro-apoptotic effects of proteasome inhibitors. We have shown previously (Schulman et al., 2019), as have others (Fernandez-Marrero et al., 2016), that deletion of just Bok from MEFs does not alter responsiveness to various triggers of apoptosis, including SST, MG132, TG, BFA and TN, suggesting that in MEFs, Bok is not a primary mediator of apoptosis, but leaving open the possibility that it might act redundantly with the *bona fide* MOMP mediators, Bak and Bax (Ke et al., 2015; Ke et al., 2018). Thus, we explored the role of Bok in Bak/Bax double (DKO) MEFs, since in these cells, it might be possible to clearly identify an apoptotic role for Bok. Interestingly, initial examination of the effects of the various triggers of apoptosis in WT or DKO MEFs (as indicated by caspase-3 cleavage) revealed that only MG132 retained activity in DKO MEFs (Figure 2A, lane 6), suggesting that Bok could potentially be contributing to the pro-apoptotic effects of MG132. However, MG132-induced caspase-3 cleavage was essentially identical in DKO and Bak/Bax/Bok triple KO (TKO) MEFs, either when examined shortly after Bok deletion in heterogeneous cultures (Figure 2B, lanes 6 vs. 8), or after the creation of clonal cell lines (Figure 2C, lanes 2 vs. 4). Cell death measurements (Figure 2D) showed that the effects of 4 h with MG132 were negligible (as expected, considering the relatively low amount of caspase-3 cleavage at that time point, Figure 2A), and that even the increased cell death seen at longer times with MG132 was still unaffected by Bok deletion. Thus, in MEFs, MG132 effects are largely Bak/Bax/Bok independent, and clearly, Bok does not mediate proteasome inhibitor-induced apoptosis.

With regard to the mechanism of MG132 action, caspase-8 cleavage was enhanced by MG132 in parallel to caspase-3 (Figures 2B,C), suggesting that MG132 may be triggering activation of the death receptor or “extrinsic” apoptosis pathway, which in turn causes caspase-3 cleavage (Tummers and Green, 2017; Carneiro and El-Deiry, 2020). Possible MOMP involvement was also assessed by measuring cytochrome c release from mitochondria, but revealed no substantial effect of MG132 (Supplementary Figure S2). Thus, MG132-induced apoptosis in MEFs appears to occur independently of MOMP, consistent with other studies (Laussmann et al., 2011; Hellwig et al., 2022).

### Endogenous Bok expression level is critically dependent on IP_3_Rs

**FIGURE 3:**
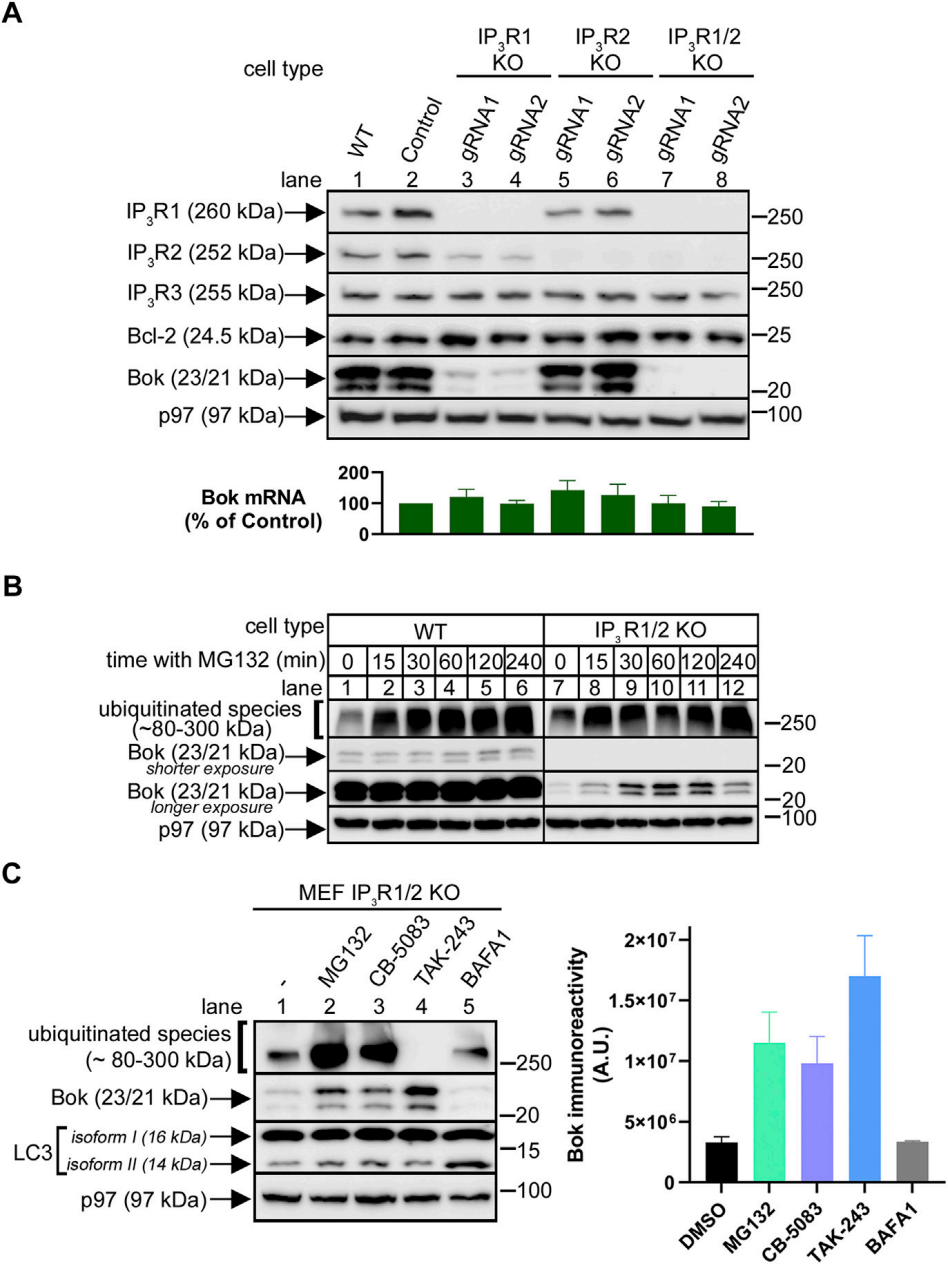
Endogenous Bok expression in MEFs is dramatically reduced by IP_3_R1 and IP_3_R2 deletion. **(A)**, Immunoreactivity of Bok and other pertinent proteins in lysates from WT, control and IP_3_R1, IP_3_R2, or IP_3_R1/2 KO MEFs, with p97 serving as a loading control. For both IP_3_R1 and IP_3_R2, two different gRNAs were used (lanes 3–8). The histogram shows Bok mRNA levels in control and IP_3_R KO MEFs, mean ± SEM, *n* = 4. **(B)**, Immunoreactivity of Bok and ubiquitinated species in lysates from WT and IP_3_R1/2 KO cells incubated with 10 µM MG132 for various times, with p97 serving as a loading control. **(C)**, Immunoreactivity of Bok and other pertinent proteins in lysates from IP_3_R1/2 KO cells incubated with 10 µM MG132, CB-5083, TAK-243, and 100 nM bafilomycin A1 (BAFA1) for 2 h, with p97 serving as a loading control. Changes in the levels of ubiquitinated species and LC3 isoform II serve as positive controls for the various drugs. The histogram shows quantitated Bok immunoreactivity, mean ± SEM, *n* = 3.

Since it has been shown previously that the stability and expression level of endogenous Bok are governed by binding to IP_3_Rs in αT3 cells and DT40 cells (Schulman et al., 2016; Schulman et al., 2019), we wondered if the same is true in MEFs, in which endogenous Bok is clearly expressed well and is not actively degraded by the proteasome (Figure 1C). Bok binds strongly to IP_3_R1 and IP_3_R2, but not to IP_3_R3, and since MEFs contain IP_3_R1-3 (Schulman et al., 2013) we individually and then consecutively targeted IP_3_R1 and IP_3_R2 using CRISPR/Cas9-mediated gene editing, and specifically deleted these proteins (Figure 3A). Remarkably, deletion of IP_3_R1 caused a major decline in Bok levels (Figure 3A, lanes 3 and 4), while deletion of IP_3_R2 had little or no effect (lanes 5 and 6), consistent with the relatively small contribution of IP_3_R2 to total MEF IP_3_R content (Schulman et al., 2013). Deletion of both IP_3_R1 and IP_3_R2 caused the most dramatic decline in Bok levels (lanes 7 and 8). Levels of Bcl-2 were unaffected, showing that the effect is specific to Bok. The expression level of Bok in IP_3_R1/2 KO MEFs was ∼2% of that seen in control cells, since the Bok immunoreactivity seen with 50 µg of IP_3_R1/2 KO cell lysate was approximately equivalent to that seen with 1 µg of WT cell lysate (Supplementary Figure S3A). The decline in Bok expression was apparently not due to a change in Bok synthesis, as Bok mRNA levels were not substantially different between WT and IP_3_R KO cell lines (Figure 3A, histogram), indicating that Bok is degraded when IP_3_R1 and IP_3_R2 are absent. The proteasome appears to be the degradation route, since treatment with MG132 caused a rapid increase in Bok levels in IP_3_R1/2 KO cells (Figure 3B, lanes 7–12), that was not seen in WT cells (lanes 1–6). In IP_3_R1/2 KO cells, Bok immunoreactivity increased ∼5-fold after 1 h with MG132 (lane 7 vs. 10), to a level ∼10% of that seen in WT cells (Supplementary Figure S3B). Further, 1 h with MG132 did not significantly alter Bok mRNA levels in IP_3_R1/2 KO MEFs (levels after MG132 treatment were 119 ± 15% of that in control cells; mean ± *n* = 5). Thus, in IP_3_R1/2 KO cells, MG132 markedly increases Bok immunoreactivity without affecting Bok mRNA levels, suggesting that newly-synthesized Bok is degraded by the proteasome when it is unable to access IP_3_R binding sites. Other classes of UPP blocking drugs also elevated Bok levels; the p97 inhibitor CB-5083 (Anderson et al., 2015) and the UBE1 inhibitor TAK-243 (Hyer et al., 2018) both increased Bok levels similarly to MG132 (Figure 3C, lanes 2–4). In contrast, the lysosome inhibitor bafilomycin A1 did not affect Bok levels (lane 5). Why Bok levels in IP_3_R1/2 KO cells are only increased to ∼10% of WT levels by UPP inhibition is presently unclear, but is most likely because UPP blockade inhibits protein synthesis (Guan et al., 2014) thus limiting Bok restoration.

Regarding the enzymes/proteins that could be mediating Bok degradation in IP_3_R1/2 KO MEFs, we examined several candidates (ube2J1, gp78, Hrd1, and erlin2) (Christianson and Carvalho, 2022) *via* CRISPR/Cas9-mediated deletion. However, none of these deletions increased Bok immunoreactivity, indicating these proteins are not involved (Supplementary Figure S4).

### IP_3_R features required for Bok stabilization

**FIGURE 4:**
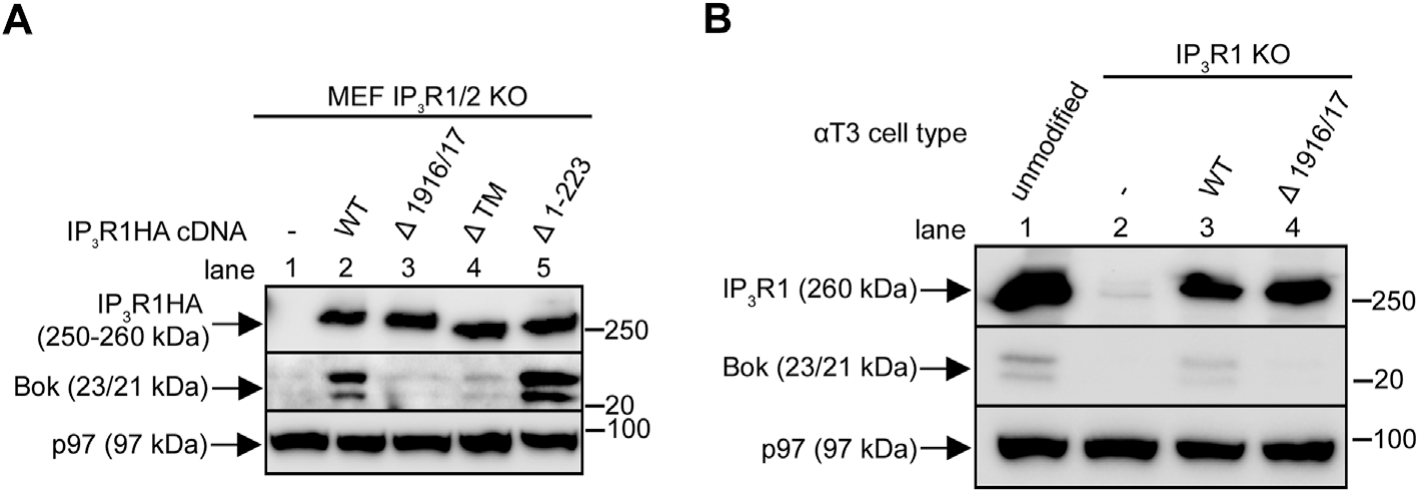
Restoration of endogenous Bok expression with exogenous IP_3_R1 constructs. **(A)**, IP_3_R1/2 KO MEFs were transiently transfected to express IP_3_R1HA constructs and lysates were probed for Bok and IP_3_R1HA constructs with anti-Bok and anti-HA, respectively, with p97 serving as a loading control. **(B)**, IP_3_R1 KO αT3 cells were transfected to stably express IP_3_R1HA^WT^ or IP_3_R1HA^Δ^ ^1916/17^ and lysates, together with lysates from unmodified αT3 cells (lane 1) were probed for Bok and for IP_3_R1HA constructs/endogenous IP_3_R1 with anti-Bok and anti-IP_3_R1, respectively, with p97 serving as a loading control.

Exogenous IP_3_R1HA constructs (Szczesniak et al., 2021a; Gao et al., 2022) were expressed transiently in IP_3_R1/2 KO MEFs (Figure 4A). IP_3_R1HA^WT^ increased endogenous Bok levels, indicating that if normal Bok binding sites are introduced, newly synthesized Bok is stabilized (lane 2 vs. 1). The specificity of this effect is demonstrated by the finding that IP_3_R1HA^Δ^ ^1916/17^, which does not bind Bok (Szczesniak et al., 2021a), did not restore Bok levels (lane 3). Likewise, IP_3_R1HA^Δ^™, which lacks TM domains 1–6 and localizes to the cytosol (Szczesniak et al., 2021a), did not restore Bok expression, indicating that events that control Bok stability occur at the ER membrane (lane 4). Finally, IP_3_R1HA^Δ1-223^, which lacks the “suppressor domain” and has no Ca^2+^ channel activity (Uchida et al., 2003), also restored Bok levels, indicating that it is the presence of ER-located Bok binding sites, rather than functional Ca^2+^ channels that stabilizes Bok (lane 5). Similar observations can be made in IP_3_R1 KO αT3 cells (Schulman et al., 2016) in which stable expression of exogenous IP_3_R1HA^WT^, but not IP_3_R1HA^Δ^ ^1916/17^, partially restores endogenous Bok expression (Figure 4B). The lack of Bok restoration with IP_3_R1HA^Δ^ ^1916/17^ is not because of impaired functionality, since IP_3_R1HA^Δ^ ^1916/17^ restores GnRH- induced Ca^2+^ mobilization in IP_3_R1 KO αT3 cells just as well as IP_3_R1HA^WT^ (Supplementary Figure S5). Overall, these data indicate that Bok is stabilized by its binding site present in membrane-bound IP_3_Rs, but without any need for IP_3_R channel activity. These conclusions were confirmed in a different cell line, IP_3_R1-3 KO HeLa cells (Ando et al., 2018), which express much less Bok than unmodified HeLa cells, and in which TAK-243 and IP_3_R1HA^WT^ (but not IP_3_R1HA^Δ^ ^1916/17^) partially restore endogenous Bok levels (Supplementary Figure S6).

### Location of endogenous Bok in the presence and absence of IP_3_Rs

**FIGURE 5:**
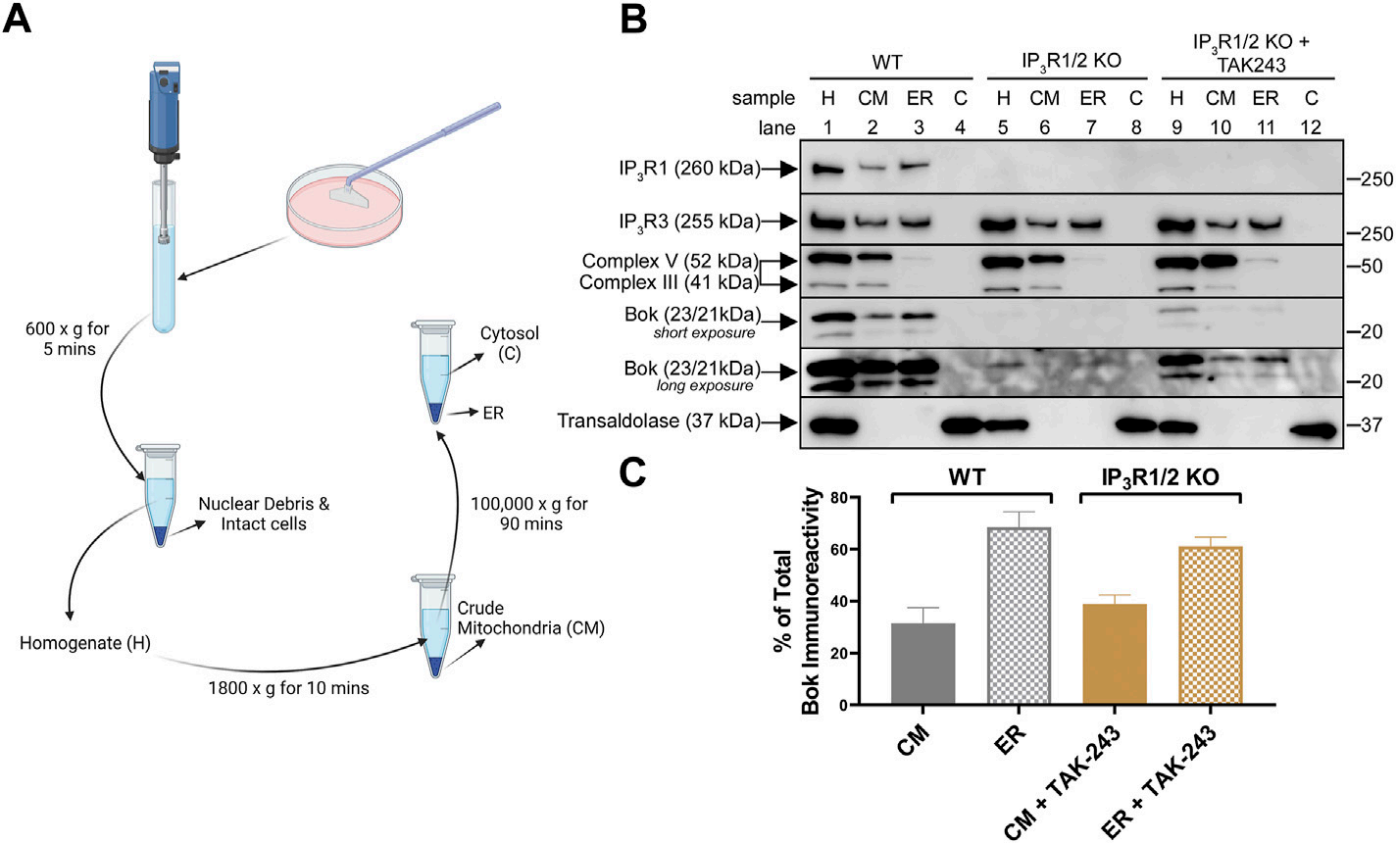
Subcellular localization of endogenous Bok. **(A)**, Summary of subcellular fractionation method created with BioRender.com. After cell disruption, centrifugation at 600 × g for 5 min produced a clear homogenate, H, free of nuclear debris and intact cells. Centrifugation of the H fraction at 1,800 × g for 10 min generated a crude mitochondria, CM, fraction (pellet) and a supernatant that was centrifuged at 100,000 × g for 90 min to generate ER (pellet) and cytosol, C, (supernatant) fractions. **(B)**, WT or IP_3_R1/2 KO MEFs, incubated without or with 10 µM TAK-243 for 1 h, were subjected to subcellular fractionation and samples were probed in immunoblots as indicated. The fraction markers used were: complex V/III for CM (inner mitochondrial membrane electron transport chain proteins), IP_3_R1 and IP_3_R3 for ER (multi-TM, ER-membrane located Ca^2+^ channels), and transaldolase for C (cytosol-located pentose phosphate pathway enzyme). Note that 1) the CM fraction contains some ER, as both IP_3_R1 and IP_3_R3 were present (lane 2), 2) complex V/III were not present in the ER and C fraction (lane 3), indicating that these fractions do not contain mitochondria, and 3) transaldolase, was only detectable in the C fraction (lane 4), indicating that cytosol was absent in both the CM and ER fractions. **(C)**, Histogram showing quantitated Bok immunoreactivity in CM and ER fractions of WT MEFs and TAK-243-treated IP_3_R1/2 KO MEFs (mean ± SEM, *n* = 2). Due to weak Bok immunoreactivity in TAK-243-treated IP_3_R1/2 KO MEFs, only the 23 kDa Bok band was quantified in all samples.

To better understand the route of Bok degradation in IP_3_R1/2 KO MEFs, subcellular fractionation was performed (Figure 5A). As expected, in WT MEFs (Figure 5B, lanes 1–4), Bok was found predominantly in the ER fraction with none in the cytosol (C) fraction, but with also some in the crude mitochondria (CM) fraction; ER:CM ratio ∼68:32 (Figure 5C). The presence of some ER in the CM fraction, as indicated by IP_3_R1 and IP_3_R3, likely accounts for the presence of Bok in the CM fraction. The residual Bok present in IP_3_R1/2 KO MEFs was not detectable using subcellular fractionation (Figure 5B, lanes 5–8), however, TAK-243 treatment revealed that stabilized Bok was predominately in the ER fraction with none in C fraction (Figure 5B, lanes 9–12) and with approximately the same distribution as that seen in WT cells; ER:CM ratio ∼61:39 (Figure 5C). Thus, in the presence or absence of Bok binding sites provided by IP_3_Rs, Bok is inserted into the ER membrane.

### Role of the atypical TM domain in Bok stability and over-expressed Bok-induced apoptosis

**FIGURE 6:**
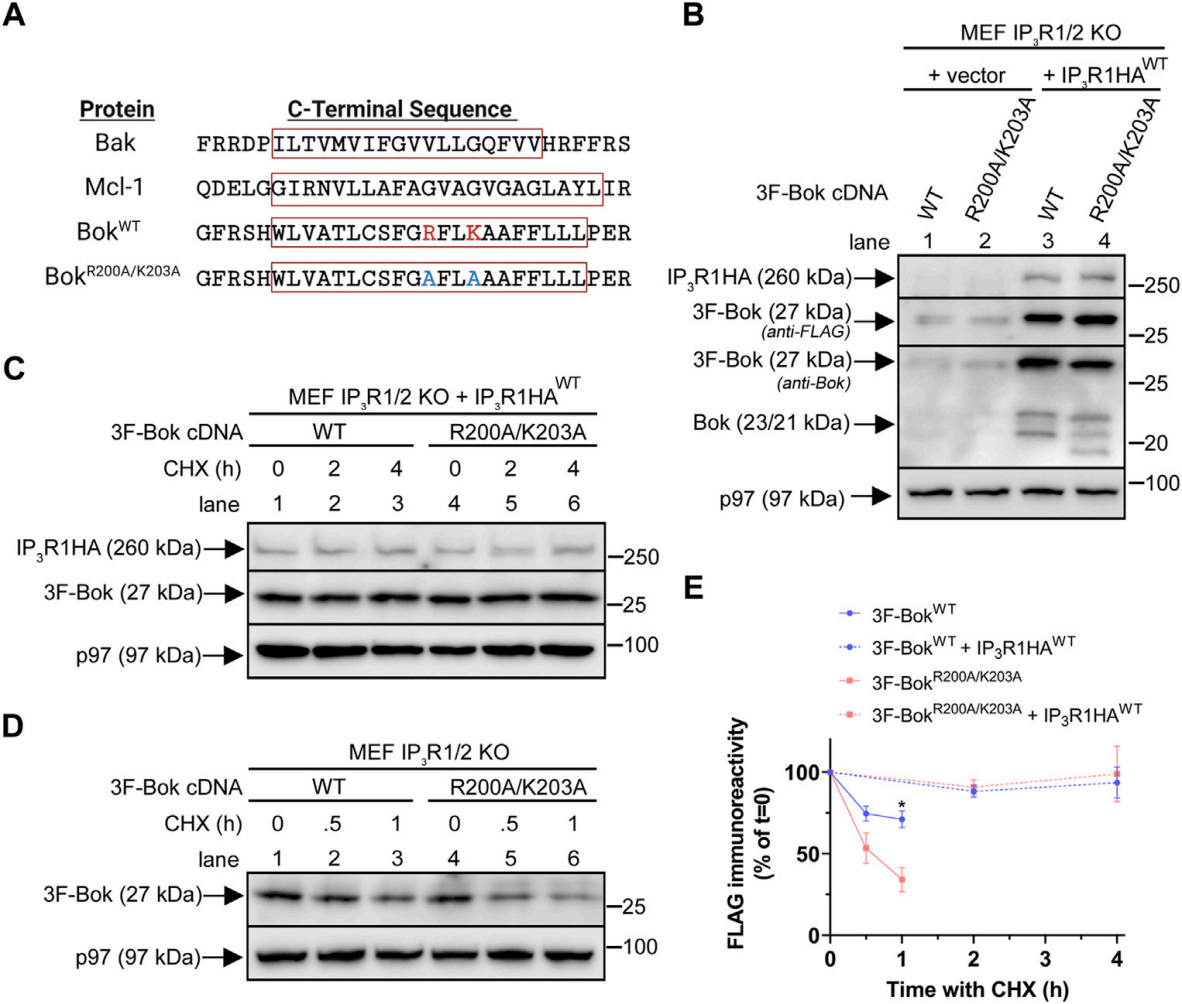
Effects of R200/K203 mutation on Bok expression in MEFs. **(A)**, C-terminal sequences of selected Bcl-2 family proteins with the TM domains enclosed in red boxes (obtained using Uniprot TM domain prediction and TMHMM and Phobius software). R200 and K203 are highlighted in red and alanine mutations are highlighted in blue. The position of the predicted TM domain was identical in both Bok^WT^ and Bok^R200A/K203A^. **(B)**, IP_3_R1/2 KO MEFs were transiently transfected with 2 µg of 3F-Bok constructs together with 8 µg of empty vector or IP_3_R1HA^WT^ and samples were probed in immunoblots as indicated. As previously described (Schulman et al., 2016), 3F-Bok^WT^ generates three Bok immunoreactive bands at 27, 23, and 21 kDa with the anti-FLAG immunoreactive band at 27 kDa corresponding to full-length 3F-Bok and the bands at 23 and 21 kDa corresponding to untagged Bok starting at Met^1^ and Met^15^. For 3F-Bok^R200A/K203A^ the lower bands were slightly shifted to approximately 22.5 and 19 kDa. **(C,D),** IP_3_R1/2 KO MEFs were transiently transfected with 2 µg of 3F-Bok constructs and 8 µg of IP_3_R1HA^WT^ **(C)** or 5 µg of 3F-Bok constructs **(D)** and cells were treated with 20 μg/ml of CHX for the times indicated. Samples were probed in immunoblots as indicated. **(E)**, FLAG immunoreactivity was quantified from **(C,D)** (% of *t* = 0, mean ± SEM, *n* = 4, * designates *p* < 0.05).

A feature of Bok unique among Bcl-2 family proteins is the presence of two charged residues (R200 and K203) within the TM domain (Figure 6A). To explore their significance, these two residues were mutated to alanine, creating 3F-Bok^R200A/K203A^ (Figure 6A). Analysis in comparison to 3F-Bok^WT^ in transiently transfected IP_3_R1/2 KO MEFs showed that the expression of both constructs was greatly enhanced by co-expression of IP_3_R1HA^WT^, indicating that both are localized to the ER membrane and stabilized by IP_3_R1 binding (Figure 6B). Further examination of stability using cycloheximide (CHX) chase showed that both constructs were turned-over very slowly in the presence of IP_3_R1HA^WT^ (Figures 6C,E), while in the absence of IP_3_R1HA^WT^, the constructs were turned-over rapidly, with 3F-Bok^R200A/K203A^ being slightly less stable than 3F-Bok^WT^ (Figures 6D,E). Thus, while R200 and K203 do not affect the ability of Bok to interact with and be stabilized by IP_3_Rs, they slightly influence the stability of non-IP_3_R-bound “free” Bok.

**FIGURE 7:**
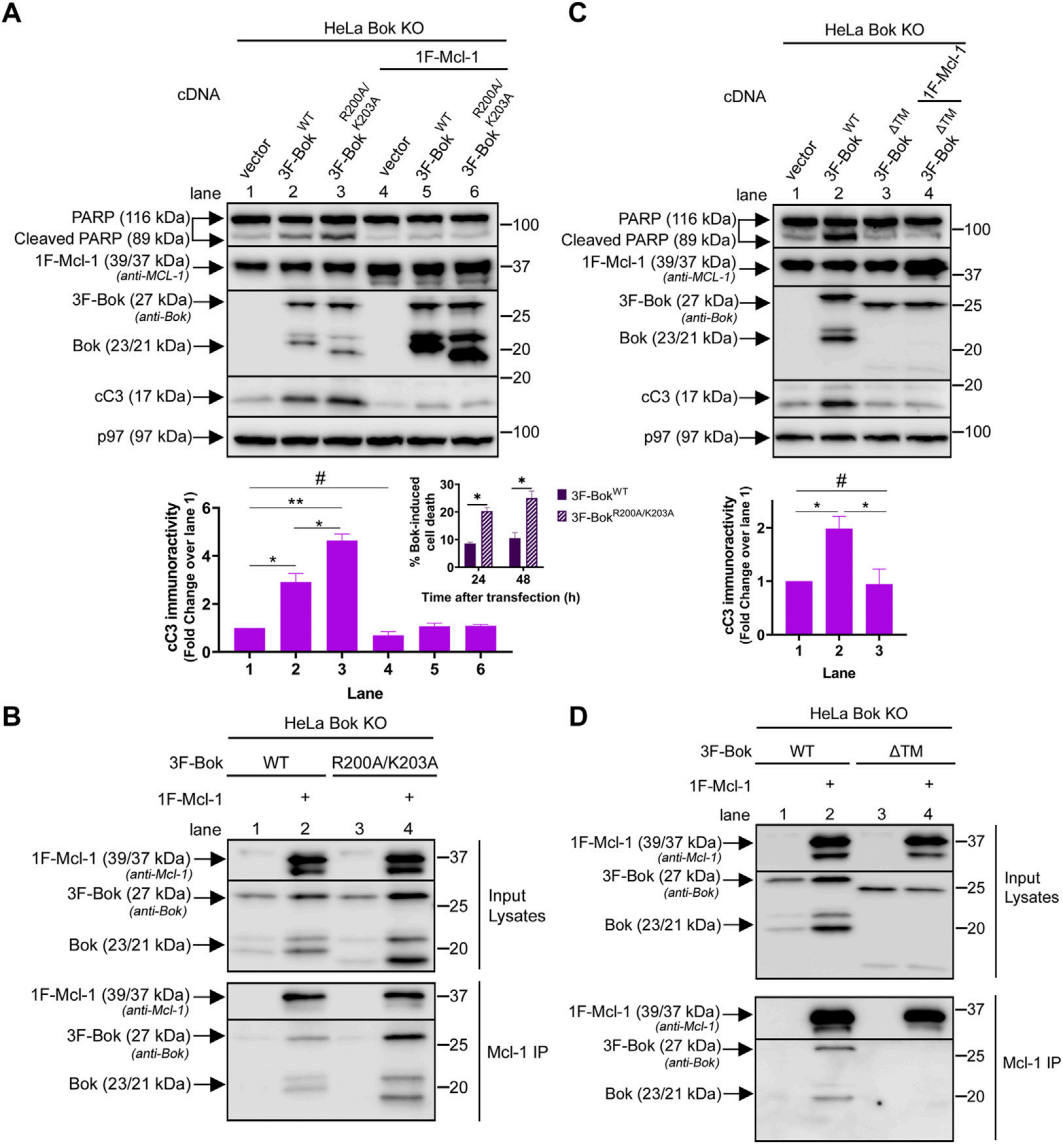
Effects of R200/K203 mutation on apoptosis induction in HeLa cells. **(A,C)**, Bok KO HeLa cells were transiently transfected with 3F-Bok constructs without or with 0.025 µg 1F-Mcl-1 and samples were probed in immunoblots as indicated with anti-Bok to identify Bok species and anti-Mcl-1, to identify both endogenous human Mcl-1 (39 kDa) and exogenous mouse 1F-Mcl-1 (39/37 kDa). The histograms show quantitated cleaved caspase-3 (cC3) immunoreactivity (mean ± SEM *n* = 3, * designates *p* < 0.05, ** designates *p* < 0.005 and # designates not significant, *p* > 0.05). The insert in panel **(A)** shows cell death induced by 3F-Bok^WT^ and 3F-Bok^R200A/K203A^, 24 and 48 h post-transfection (mean ± SEM *n* = 2, * designates *p* < 0.05). **(B,D)**, Bok KO HeLa cells were transiently transfected with 3F-Bok constructs without or with 0.5 µg 1F-Mcl-1, anti-Mcl-1 IPs were performed and samples were probed in immunoblots as indicated. 3F-Bok^Δ^™ generates Bok immunoreactive bands at ∼25 and 18 kDa.

To investigate how R200 and K203 might impact the ability of over-expressed Bok to trigger apoptosis, we utilized Bok KO HeLa cells (Szczesniak et al., 2021b), since in this cell type exogenous Bok can induce apoptosis, most likely due to high transfection efficiency and high Bok over-expression levels (Schulman et al., 2016; Chernousova and Epple, 2017; Szczesniak et al., 2021b). Intriguingly, 3F-Bok^R200A/K203A^ caused significantly more apoptosis than 3F-Bok^WT^, as indicated by caspase-3 and PARP cleavage, as well as cell death (Figure 7A, lanes 1–3, blot, histogram and insert). As exogenous Bok and anti-apoptotic Mcl-1 interact (Lucendo et al., 2020; Szczesniak et al., 2021b), we examined the effects of Mcl-1 co-expression and found that Mcl-1 blocked the apoptotic effects of both 3F-Bok^WT^ and 3F-Bok^R200A/K203A^ while, surprisingly, strongly enhancing their expression (Figure 7A, lanes 4–6, blot and histogram). Consistent with these findings, both constructs co-immunoprecipitated with exogenous Mcl-1, with 3F-Bok^R200A/K203A^ interacting slightly more strongly than did 3F-Bok^WT^ (Figure 7B, lane 4 vs. 2). These data suggest that 3F-Bok^R200A/K203A^ may exhibit enhanced pro-apoptotic activity because it binds to and antagonizes the anti-apoptotic activity of Mcl-1 better than 3F-Bok^WT^. To validate this idea, we examined 3F-Bok^Δ^™ since the interaction between Bok and Mcl-1 is dependent on the Bok TM domain (Lucendo et al., 2020; Szczesniak et al., 2021b; Sancho and Orzaez, 2021). Indeed, 3F-Bok^Δ^™ did not trigger apoptosis (Figure 7C, lane 3), or interact with exogenous Mcl-1 (Figure 7D, lane 4), and was not stabilized by Mcl-1 co-expression (Figures 7C,D). Overall, these data show that the Bok TM domain is required for apoptotic signaling and the interaction with Mcl-1, while R200 and K203 of Bok may tune its ability to network with Mcl-1 and influence apoptosis.

## Discussion

By carefully examining the properties of endogenous Bok we have gained insight into its stability and biological role. Most importantly, we find that endogenous Bok is readily-detectable in all cell types examined and is not actively degraded by the UPP, that it does not mediate the pro-apoptotic effects of proteasome inhibitors, and that Bok expression level is critically dependent upon the binding sites provided by IP_3_Rs, since when IP_3_Rs are deleted, Bok is rapidly degraded at the ER membrane by the UPP. Further, using exogenous Bok constructs we find that the atypical charged residues in the TM domain of Bok regulate its stability and when over-expressed, its pro-apoptotic activity.

Our investigation into the expression level and stability of endogenous Bok was prompted by reports that Bok is undetectable in certain cell types (e.g., MEFs), that its levels are greatly increased by proteasome inhibitors, which in turn causes apoptosis, and the ensuing hypothesis that endogenous Bok is kept at very low (“safe”) levels because it is actively degraded by the UPP (Llambi et al., 2016; Moldoveanu and Zheng, 2018; Zheng et al., 2018; Muenchow et al., 2020). Our data are not consistent with these findings, since endogenous Bok was readily-detectable in all cell types examined (including MEFs) and its levels were not increased by incubation with proteasome inhibitors. Many other studies have also shown that Bok is expressed at readily-detectable levels in various types of cells; e.g., HepG2 (Srivastava et al., 2019), 16HBE (Zhang et al., 2019), H292 (Yang et al., 2022), SH-SY5Y (Di et al., 2021; Walter et al., 2022), and that proteasome inhibitors do not increase Bok levels (Carpio et al., 2015; Schulman et al., 2016). Further, we did not observe a stabilizing effect of mutation of the six lysine residues in Bok, indicating that even exogenous Bok is not degraded by the canonical, lysine ubiquitination-dependent UPP. Finally, deletion of Bok from either wild-type MEFs or Bak/Bax DKO MEFs did not alter proteasome inhibitor-induced apoptosis, indicating that endogenous Bok does not mediate that process. Thus, overall, we conclude that endogenous Bok is relatively stable, is not actively degraded by the UPP, and does not mediate proteasome inhibitor-induced apoptosis.

Rather, the major factor governing the expression level of endogenous Bok in MEFs is the presence of Bok binding sites provided by IP_3_Rs. This was revealed by the fact that Bok levels collapse to ∼ 2% of control when IP_3_R1 and IP_3_R2 are deleted from MEFs, and by partial recovery of Bok levels when these cells are reconstituted with exogenous IP_3_R1s that contain the intact Bok binding site (the loop between residues 1882 and 1957). This partial recovery was also seen with an IP_3_R1 mutant that lacks Ca^2+^ channel activity, but not by a cytosol-located IP_3_R1 mutant, indicating that it is the presence of ER membrane-located Bok binding sites provided by IP_3_Rs, rather than functional Ca^2+^ channels, that stabilizes Bok. Surprisingly, UPP inhibitors partially restored Bok levels in IP_3_R1/2 KO MEFs, indicating that in the absence of binding sites provided by IP_3_Rs, Bok can actually be degraded by the UPP. More specifically, it appears that the ER-associated degradation (ERAD) pathway (Christianson and Carvalho, 2022) is responsible, since subcellular fractionation showed that endogenous Bok is always ER membrane-associated, either in wild-type MEFs, or in UPP inhibitor-treated IP_3_R1/2 KO MEFs, where the residual Bok accumulates. Thus, our data suggest that in MEFs, newly-synthesized Bok is inserted into the ER membrane and is stable if binding sites provided by IP_3_Rs are present; otherwise, the inserted Bok is rapidly degraded by the ERAD pathway. These conclusions are likely universal, as endogenous Bok exhibited the same characteristics in other cell types (αT3 IP_3_R1 KO and HeLa IP_3_R1-3 KO cells). Interestingly, the situation for Bok is not unique, as there are other examples of a collapse in the levels of an ER membrane protein upon deletion of a binding partner; e.g., Hrd1 levels decrease upon deletion of Hrd3/Sel1L (Sun et al., 2014; Vashistha et al., 2016).

As yet, we have been unable to identify the enzymes that mediate endogenous Bok ERAD, since KO of several of the major players in mammalian ERAD (Christianson and Carvalho, 2022) did not increase Bok levels in IP_3_R1/2 KO MEFs. These included gp78 and erlin2, which were shown previously to mediate the ERAD of exogenous Bok (Llambi et al., 2016). Thus, it appears that endogenous and exogenous Bok are degraded by different enzymes. A related question is which proteins allow for endogenous Bok to be delivered to and inserted into the ER membrane. Bok, like many other Bcl-2 family members, is a tail-anchored (TA) protein; i.e., its TM domain is found very close to the C-terminus (Popgeorgiev et al., 2018). In contrast to more typical membrane proteins, TA proteins utilize non-canonical mechanisms to allow for membrane insertion (Chio et al., 2017; Farkas and Bohnsack, 2021), yet these mechanisms for Bok, or Bcl-2 family members in general, have yet to be established. Resolution of these questions may come from conventional mass spectrometry analysis of proteins that co-purify with endogenous Bok, or perhaps, proximity labeling (Coyaud et al., 2015; Szczesniak et al., 2021b).

Even though it appears that all endogenous Bok is bound to IP_3_Rs (Schulman et al., 2016), it is intriguing that several studies indicate that exogenous Bok can trigger apoptosis (Hsu et al., 1997; Llambi et al., 2016; Zheng et al., 2018) and that exogenous Bok and Mcl-1 bind and that this interaction is mediated by their TM domains (Lucendo et al., 2020; Szczesniak et al., 2021b; Sancho and Orzaez, 2021). While examining the role of the atypical, charged residues (R200 and K203) in the Bok TM domain, we found that their mutation to uncharged and more hydrophobic alanine enhanced apoptotic signaling, and also that Mcl-1 bound to and blocked the pro-apoptotic effects of Bok constructs, despite strongly enhancing their expression. Thus, there is clearly interplay between Bok and Mcl-1, with the R200 and K203 residues apparently tuning that interaction. Whether this translates to endogenous proteins is unclear, particularly as interaction between endogenous Bok and Mcl-1 is not readily-detectable (Lucendo et al., 2020; Szczesniak et al., 2021b). Indeed, the pro-apoptotic effects of over-expressed Bok may not be physiologically relevant since non-IP_3_R-bound “free” Bok could artificially cause MOMP directly, or indirectly by perturbing the Bcl-2 family network (Kale et al., 2018; Xiang et al., 2018). Thus, while we show that exogenous, over-expressed Bok can induce apoptosis, we find no evidence that endogenous Bok does the same.

Overall, our data indicate that endogenous Bok is an ER membrane protein whose stability is critically dependent upon being able to bind IP_3_Rs. As all endogenous Bok appears to be IP_3_R-bound and deletion of endogenous Bok has no effect on apoptotic signaling in our studies, it seems unlikely that endogenous Bok is a significant contributor to MOMP and apoptosis, and that it will be productive to investigate novel, non-apoptotic roles of this intriguing protein.

## Data Availability

The raw data supporting the conclusion of this article will be made available by the authors, without undue reservation.

## Abbreviations

Bok, Bcl-2-related ovarian killer; cC3, cleaved caspase-3; ER, endoplasmic reticulum; ERAD, ER-associated degradation; HEK293T, human embryonic kidney; HCT116, human colon carcinoma; IP, immunoprecipitation; IP_3_R, inositol 1,4,5-trisphosphate receptor; KO, knock-out; MAMs, mitochondria-associated membranes; MEF, mouse embryonic fibroblast; αT3, mouse pituitary cells; MOMP, mitochondrial outer membrane permeabilization; Mcl-1, myeloid-cell leukemia 1; TM, transmembrane; UPP, ubiquitin-proteasome pathway.
