# Supplementary material for: Endogenous Bok is stable at the endoplasmic reticulum membrane and does not mediate proteasome inhibitor-induced apoptosis
Source: Front Cell Dev Biol. 2022 Dec 19;10:1094302. doi: 10.3389/fcell.2022.1094302 (PMC9806350; doi:10.3389/fcell.2022.1094302)
Supplement: Supplementary file 1 [file DataSheet7.PDF]

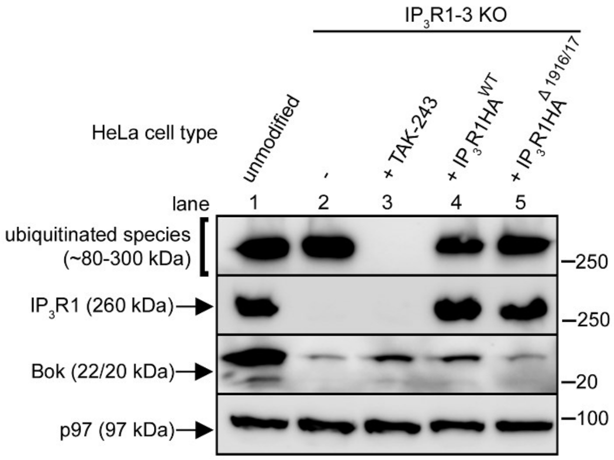

Supplementary Figure 6. Restoration of endogenous Bok expression in IP_3R1-3 KO HeLa cells. Immunoreactivity of Bok and other pertinent proteins in unmodified (lane 1) and IP_3R1-3 KO HeLa cells under various conditions (lanes 2-5). Endogenous Bok levels are dramatically reduced in IP_3R1-3 KO cells compared to unmodified HeLa cells (lanes 2 and 1, respectively) and Bok immunoreactivity is partially restored by treatment with 10 μM TAK-243 for 1.5 h (lane 3) and exogenous IP_3R1HA^{WT} (lane 4), but not by $IP_3R1HA^{\Delta 1916/17}$ (lane 5). Lysates were probed for Bok and for IP_3R1HA constructs/endogenous IP_3R1 with anti-Bok and anti-IP_3R1, respectively, with p97 serving as a loading control and ubiquitinated species serving as a positive control for TAK-243 effectiveness.
