# Supplementary material for: Endogenous Bok is stable at the endoplasmic reticulum membrane and does not mediate proteasome inhibitor-induced apoptosis
Source: Front Cell Dev Biol. 2022 Dec 19;10:1094302. doi: 10.3389/fcell.2022.1094302 (PMC9806350; doi:10.3389/fcell.2022.1094302)
Supplement: Supplementary file 2 [file DataSheet2.PDF]

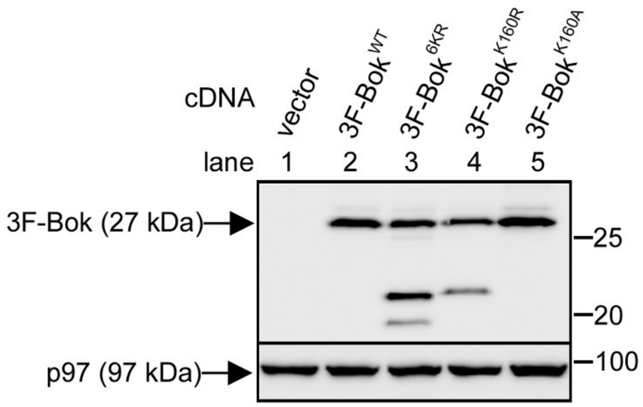

Supplementary Figure 1. Mutation of K160 to arginine causes exogenous Bok fragmentation. HEK293T cells were transiently transfected to express mouse 3F-Bok constructs and cell lysates were probed as indicated with anti-FLAG, with p97 serving as a loading control. The constructs used were either 3F-Bok^{WT}, 3F-Bok^{6KR} (all 6 lysines in Bok mutated to arginine), 3F-Bok^{K160R} (lysine 160 mutated to arginine), or 3F-Bok^{K160A} (lysine 160 mutated to alanine). As described previously (Schulman et al., 2016), the 3F-Bok^{WT} construct (lane 2) generates an anti-FLAG immunoreactive band at 27 kDa, corresponding to full length 3F-Bok. The 3F-Bok^{6KR} construct is partially fragmented, as indicated by partial loss of the 27 kDa band and appearance of bands corresponding to N-terminal regions of Bok, predominantly at ~22 and 19 kDa (lane 3). This fragmentation was largely due to mutation of lysine 160 to arginine, since similar fragmentation was seen for 3F-Bok^{K160R} (lane 4), but not for 3F-Bok^{K160A} (lane 5). Why introduction of arginine at position 160 should lead to Bok cleavage is presently unclear.

References

Schulman, J.J., Wright, F.A., Han, X., Zluhan, E.J., Szczesniak, L.M., and Wojcikiewicz, R.J. (2016). The Stability and Expression Level of Bok Are Governed by Binding to Inositol 1,4,5-Trisphosphate Receptors. *J Biol Chem* 291(22), 11820-11828. doi: 10.1074/jbc.M115.711242.
