# Supplementary material for: Endogenous Bok is stable at the endoplasmic reticulum membrane and does not mediate proteasome inhibitor-induced apoptosis
Source: Front Cell Dev Biol. 2022 Dec 19;10:1094302. doi: 10.3389/fcell.2022.1094302 (PMC9806350; doi:10.3389/fcell.2022.1094302)
Supplement: Supplementary file 3 [file DataSheet4.PDF]

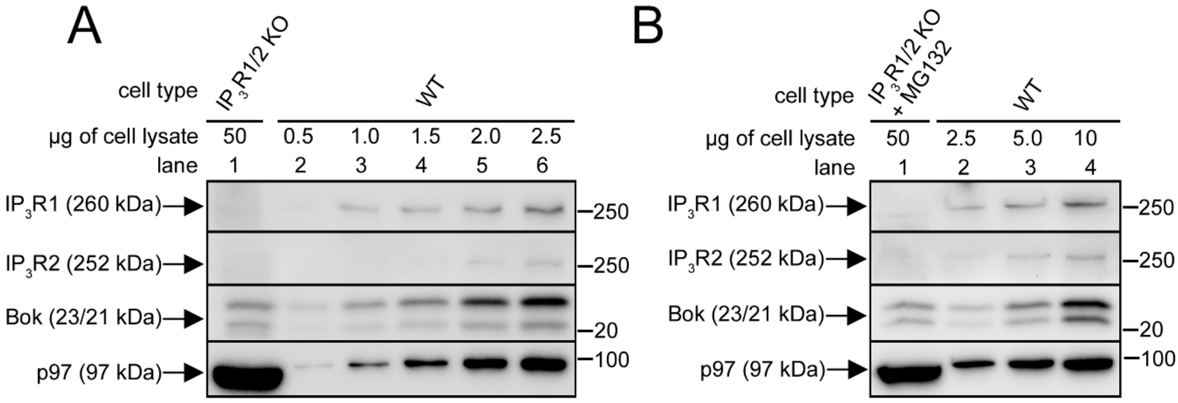


**Supplementary Figure 3. Estimation of Bok expression levels in $IP_3R1/2$ KO MEFs obtained by loading different amounts of cell lysate. A,** Comparison of Bok immunoreactivity in WT and $IP_3R1/2$ KO MEFs. Bok immunoreactivity seen with 50 µg of $IP_3R1/2$ KO cell lysate (lane 1) was approximately equivalent to that seen with 1 µg of WT cell lysate (lane 3) indicating that Bok levels in $IP_3R1/2$ KO cells are ~2% of that seen in WT cells. **B,** Comparison of Bok immunoreactivity in WT MEFs and $IP_3R1/2$ KO MEFs treated for 1 h with MG132. Bok immunoreactivity seen with 50 µg of $IP_3R1/2$ KO + MG132 cell lysate (lane 1) was approximately equivalent to that seen with 5 µg of WT cell lysate (lane 3) indicating Bok is restored to ~10% of WT levels with MG132.
