# Supplementary material for: Endogenous Bok is stable at the endoplasmic reticulum membrane and does not mediate proteasome inhibitor-induced apoptosis
Source: Front Cell Dev Biol. 2022 Dec 19;10:1094302. doi: 10.3389/fcell.2022.1094302 (PMC9806350; doi:10.3389/fcell.2022.1094302)
Supplement: Supplementary file 4 [file DataSheet6.PDF]

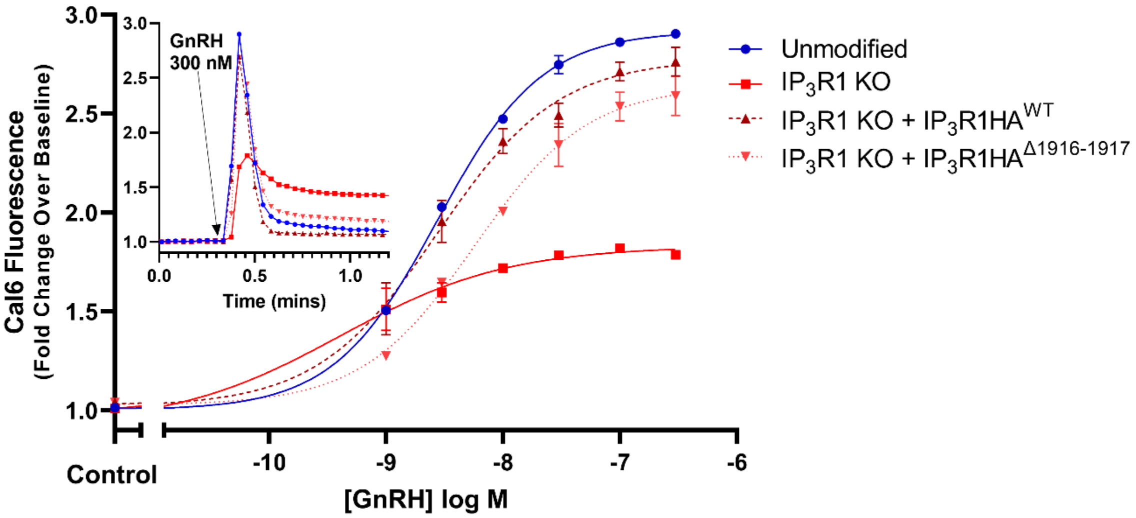

Supplementary Figure 5. Ca^{2+} mobilization in αT3 cell lines. Gonadotropin-releasing hormone (GnRH)-mediated Ca^{2+} mobilization in unmodified αT3 cells or IP_3R1 KO αT3 cells without or with stably expressed IP_3R1HA constructs. Cells were seeded into 96-well plates and the following day were subjected to the Cal6-based FLIPR Calcium Assay (Molecular Devices). Cells were exposed to a range of GnRH doses (1 nM – 300 nM) using a FlexStation3 (Molecular Devices) and Cal6 Fluorescence at 485 nm was measured (Szczesniak et al., 2021a). Shown are the dose-dependence of peak responses (mean ± SEM, n=2) and the temporal response to the maximum concentration of GnRH (representative independent experiment).

References

Szczesniak, L.M., Bonzerato, C.G., Schulman, J.J., Bah, A., and Wojcikiewicz, R.J.H. (2021a). Bok binds to a largely disordered loop in the coupling domain of type 1 inositol 1,4,5-trisphosphate receptor. *Biochem Biophys Res Commun* 553**,** 180-186. doi: 10.1016/j.bbrc.2021.03.047.
