# Supplementary material for: Endogenous Bok is stable at the endoplasmic reticulum membrane and does not mediate proteasome inhibitor-induced apoptosis
Source: Front Cell Dev Biol. 2022 Dec 19;10:1094302. doi: 10.3389/fcell.2022.1094302 (PMC9806350; doi:10.3389/fcell.2022.1094302)
Supplement: Supplementary file 5 [file DataSheet3.PDF]

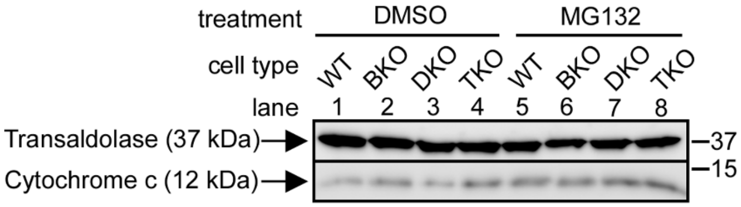

Supplementary Figure 2. Lack of MG132-induced cytochrome c release in MEFs. Heterogenous MEFs were seeded at 3 x 10^5/9.6 cm^2 well and 24 h later were treated with either DMSO (vehicle) or 10 μM MG132 for 4 h. Digitonin-induced plasma membrane permeabilization to allow for measurement of released cytochrome c from mitochondria was performed essentially as described (Heimer et al., 2019) with all steps at 4°C. Cells were harvested with PBS and centrifuged at 1000 x g for 2 minutes. Cell pellets were permeabilized with 100 μL of 190 μg/mL digitonin in 75 mM NaCl, 1 mM NaH_2PO_4, 8 mM Na_2HPO_4, 250 mM sucrose, 10 μM pepstatin, 0.2 mM phenylmethylsulfonyl fluoride, 1 mM dithiothreitol, and 0.2 μM soybean trypsin inhibitor, pH 7.5, for 10 minutes. Permeabilized cells were then centrifuged at 14,000 x g for 5 minutes and 75 μL of the supernatant (soluble or cytosolic proteins) was collected and subjected to immunoblotting, with transaldolase serving as a positive control for plasma membrane permeabilization.

References

Heimer, S., Knoll, G., Schulze-Osthoff, K., and Ehrenschwender, M. (2019). Raptinal bypasses BAX, BAK, and BOK for mitochondrial outer membrane permeabilization and intrinsic apoptosis. *Cell Death Dis* 10(8), 556. doi: 10.1038/s41419-019-1790-z.
