# Supplementary material for: Endogenous Bok is stable at the endoplasmic reticulum membrane and does not mediate proteasome inhibitor-induced apoptosis
Source: Front Cell Dev Biol. 2022 Dec 19;10:1094302. doi: 10.3389/fcell.2022.1094302 (PMC9806350; doi:10.3389/fcell.2022.1094302)
Supplement: Supplementary file 6 [file DataSheet1.PDF]

Supplementary Table 1. gRNA sequences used for CRISPR/Cas9-mediated deletion

Target	gRNA 1	gRNA 2
Bok	ACGCGCGGCTTTTGCGCGC	CCCCGCGGCCACGGAATAC
IP_3R1	GGTGCGGAGTATCGATTCAT	GCACCTCCACGCAGAGTCGT
IP_3R2	GTAACCTCTTGTTCACCGTC	CAAGTGCCTTAACCGCACGT
ube2J1	GAGTACCCCATGAAACCACC	N/A
gp78	GGATGTGGTGCATGAGGTCC	N/A
erlin2	GAGATGGAAACCCGGGCCAC	N/A
Hrd1	GTCATCCCGAAAAACGGTGA	N/A
