# Supplementary material for: Endogenous Bok is stable at the endoplasmic reticulum membrane and does not mediate proteasome inhibitor-induced apoptosis
Source: Front Cell Dev Biol. 2022 Dec 19;10:1094302. doi: 10.3389/fcell.2022.1094302 (PMC9806350; doi:10.3389/fcell.2022.1094302)
Supplement: Supplementary file 7 [file DataSheet5.PDF]

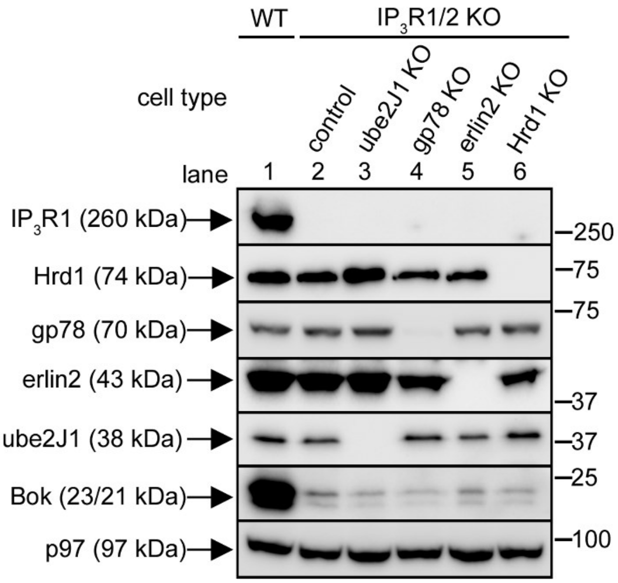

Supplementary Figure 4. Deletion of candidate mediators of Bok degradation. The ubiquitin-conjugating enzyme, ube2J1, and the ubiquitin-ligases, Hrd1 and gp78, (lanes 3, 4, and 6, respectively), as well as erlin2 (lane 5), were deleted using CRISPR/Cas9 in $IP_3R1/2$ KO MEFs. Cell lysates were probed for Bok and other pertinent proteins, with p97 serving as a loading control. Surprisingly, none of the deletions elevated Bok levels compared to control cells (lane 2), suggesting that these candidate mediators are not involved in the degradation of endogenous Bok.
